# The status of medical laboratory towards of AFRO-WHO accreditation process in government and private health facilities in Addis Ababa, Ethiopia

**DOI:** 10.11604/pamj.2015.22.136.7187

**Published:** 2015-10-14

**Authors:** Eyob Abera Mesfin, Bineyam Taye, Getachew Belay, Aytenew Ashenafi

**Affiliations:** 1 Quality Africa Network (Pty) /GIZ, Addis Ababa, Ethiopia; 2 Addis Ababa University College of Health Science, School of Medicine Addis Ababa, Ethiopia; 3 Ethiopian Health and Nutrition Research Institutes, Addis Ababa, Ethiopia; 4 African Society for Laboratory Medicine (ASLM) Addis Ababa, Ethiopia

**Keywords:** Accreditation, laboratory, quality, SLMTA, WHO-AFRO

## Abstract

**Introduction:**

The World Health Organization Regional Office for Africa (WHO AFRO) introduces a step wise incremental accreditation approach to improving quality of laboratory and it is a new initiative in Ethiopia and activities are performed for implementation of accreditation program.

**Methods:**

Descriptive cross sectional study was conducted in 30 laboratory facilities including 6 laboratory sections to determine their status towards of accreditation using WHO AFRO accreditation checklist and 213 laboratory professionals were interviewed to assess their knowledge on quality system essentials and accreditation in Addis Ababa Ethiopia.

**Results:**

Out of 30 laboratory facilities 1 private laboratory scored 156 (62%) points, which is the minimum required point for WHO accreditation and the least score was 32 (12.8%) points from government laboratory. The assessment finding from each section indicate that 2 Clinical chemistry (55.2% & 62.8%), 2 Hematology (55.2% & 62.8%), 2 Serology (55.2% & 62.8%), 2 Microbiology (55.2% & 62.4%), 1 Parasitology (62.8%) & 1 Urinalysis (61.6%) sections scored the minimum required point for WHO accreditation. The average score for government laboratories was 78.2 (31.2%) points, of these 6 laboratories were under accreditation process with 106.2 (42.5%) average score, while the private laboratories had 71.2 (28.5%) average score. Of 213 respondents 197 (92.5%) professionals had a knowledge on quality system essentials whereas 155 (72.8%) respondents on accreditation.

**Conclusion:**

Although majority of the laboratory professionals had knowledge on quality system and accreditation, laboratories professionals were not able to practice the quality system properly and most of the laboratories had poor status towards the WHO accreditation process. Thus government as well as stakeholders should integrate accreditation program into planning and health policy.

## Introduction

Quality laboratory service is an essential part of a health care delivery for diagnosis and monitoring of disease. But due to lack of awareness on the role of laboratory service in many developing countries, laboratory services have shortage of resource, poor management, ineffective services [1, 2], low quality control measures, absence quality assurance programs, shortages of training and poor staff motivation [3]. In 2008 the World Health Organization Regional Office for Africa (WHO AFRO) introduces a stepwise accreditation approach to improving quality of laboratory in Africa. The program uses an incremental stepwise approach that is objectively measurable over time using international standards and the standards were adapted to the local environment [4]. The establishment of accreditation scheme will help to improve quality of laboratory services. The accreditation is provided in a five star tiered approach and laboratories achieve less than 55% will not be awarded a star ranking [5]. Ethiopian Federal Ministry of Health supports to implement the WHO-AFRO Laboratory Accreditation Program in Ethiopia and Ethiopian Health &Nutrition Research Institute (EHNRI) has taken the task to lead accreditation activities efforts in country and established Accreditation Steering Committee to facilitate the implementation of the accreditation scheme [6]. As part of the accreditation activities EHNRI, CDC and partners conducted a baseline assessment in 24 laboratories(6 national, 7 regional and 11 hospital laboratories), in August 2010 using the WHO-AFRO Checklist and assessment results showed that 2 laboratories from EHNRI National Reference scored the minimum requirement for “1 Star” (55 & 59%). More over major identified gaps were lack of a quality manual, and documents, poor documentation of records, absence of system for evaluating client satisfaction, routine calibration of equipment, internal audit, quality control results monitoring, and corrective action [6]. In Ethiopia the laboratory infrastructure and quality assurance activities remain weak [7] and few laboratories are accredited. Moreover there is a little information available on the status of laboratories towards of accreditation process. Therefore, this study was conducted to determine the status of laboratories towards of accreditation process and to provide baseline information to policy and decision makers for improvement of laboratory quality in future.

## Methods

**Study Design:** a descriptive cross sectional study was conducted using checklist and questionnaire to determine the status of laboratories towards accreditation process and the knowledge of laboratory professionals on quality system essential elements & accreditation in government and private health facilities in Addis Ababa city.

**Study Area and Period:** the study was conducted in Addis Ababa which is the capital city of Ethiopia between December 2013 and February 2014. The city is divided into 10 sub cities and 116 Woreda [8]. The city had 33 hospitals, 34 health centers, 468 private clinics and 93 institutional based clinics. Of which, 5 hospitals were under Addis Ababa Health Bureau, 4 were under Ministry of Health referral Hospitals, 3 hospitals were uniformed forces and 1 hospital under Addis Ababa University; and 20 hospitals were private facilities [9].

**Source Population and Study Subjects:** all health facility laboratories and laboratory professionals in the city of Addis Ababa were source population for the assessment of status of the laboratories towards accreditation process and determination of their knowledge on quality system essential elements & accreditation respectively. The study subjects were health facility laboratories working more than a year and laboratory professionals who had more than a year experience in the selected health facility laboratories.

**Inclusion and Exclusion Criteria:** all laboratory professionals and health facility laboratories having more than a year experience were included in the study. Laboratory professionals and health facility laboratories which were not willing to participate in the study were excluded

**Sampling Procedures:** purposive sampling technique was used for determination of the status of laboratory towards accreditation process. All 33 hospitals’ laboratories found in Addis Ababa city were selected for the study of which 14 laboratories were laboratories from government health facilities and the remaining laboratories were from private health facilities, however the study was conducted on 30 health facility laboratories because of exclusion criteria. And a single population proportion formula was used for determination of the sample size for interviewing laboratory professionals on quality system essential elements & accreditation considering the following assumptions: Proportion of 50% was taken due to absence of reliable previous study that shows the laboratory professionals knowledge on quality system and accreditation. Level of significance = 0.05, Marginal of error (d) = 5%, Sample size = n, Z (_α/2_) = Z-score at 95% confidence interval = 1.96. The formula for calculating the sample size (n) was as follow: N = Z_α/2_^2^P(1-p)/d^2^; n = 1.96^2^*0.5*0.5/0.05^2^ = 384; then n = 384. According to Health and Health Related Indicators there were 393 laboratory professionals in Addis Ababa [9], therefore, correction factor was done using the finite population formula (nf) from a target population (N) and the sample size was reduced according to the following formula: Nf= n/n/1 + n/N; nf= 384/384/1 + 384/393; then, nf = 194. Considering 10% of non-response rate the sample size for laboratory professionals was 213. Finally sample size was allocated proportionately for each facility laboratory according to number of laboratory professionals working in the laboratories and they were selected for the interview using random sampling method. Therefore, the laboratory professionals were sampled for interview from 30 health facility laboratories.

**Data Collection Procedures:** a WHO-AFRO's accreditation checklist and questionnaire were used for data collection.

**Checklist:** a WHO-AFRO Accreditation checklist was used to assess of the status of participant laboratories towards accreditation. Assessment was conducted in six major laboratory sections/departments and on the whole laboratory facility, the major sections were Clinical chemistry, Hematology, Serology, Parasitology, Urinalysis and Microbiology Laboratories. The checklist's 12 sections provide assessment on the basis of 110 clauses and 250 total possible points. Each item has been assigned a weighted value of 2, 3, or 5 points based on complexity and/or relative importance. Incomplete fulfillment of an item can be scored as “partial” and awarded a single point, with written explanation. Some clauses in the checklist are “tick lists” and require the satisfactory presence of all sub items listed below the main heading to receive full credit [10, 11]. The checklist was focusing on assessing the quality system essentials; documents & records, management reviews, organization & personnel, client management & customer service, equipment, internal audit, purchasing & inventory, information management, process control and internal & external quality assessment, corrective action, occurrence / incident management & process improvement and facilities & safety. According to WHO-AFRO Accreditation approach the minimum score for the 1 star level is 138-160 (55%-64%) points, 2 stars level is 161-185 (65%-74%) points, 3 stars level is 186-211(75%-84%) points, 4 stars level is 212 -236 (85%-94%) points and 5 stars level is 237-250 points (95% -100%) points of the standard.

**Questionnaire:** the data collection instrument interviewed by data collectors were anonymously closed ended questionnaire. It included different questions, education background, characteristics of laboratory professionals, work experience, and regarding knowledge and attitude of laboratory quality system essential elements and accreditation. Three trained and senior laboratory technologists assessed the laboratory status for accreditation using the WHO AFRO checklist and they interviewed laboratory professionals using questionnaire for data collection. Principal investigator involved in overall controlling activities of data collections and assisted data collectors during the process of data collection and collected filled questionnaires regularly and checked for inconsistencies and omission.

**Data Management and Statistical Analysis:** the collected data were cleaned, coded, fed in to SPSS version 16 statistical software by principal investigator and data clerk. After entry, the data were re-cleaned to correct errors and they were also categorized as necessary. The data entered were checked for consistency, moreover frequencies and cross tabulations were used to check missed values and variables. Descriptive statistics were computed to calculate the percentage of each laboratory score and the responses of the laboratory professionals.

**Data Quality Assurance:** to assure data quality, data collectors were trained for two days and the questionnaire was pre-tested before the actual data collection. Completeness, accuracy and consistency of the collected data were checked on daily bases during data collection by the principal investigator, where those questionnaires found incomplete, inaccurate and inconsistent was returned back for data collectors to be filled again. Data were cleaned, edited and coded before data entry and then recoded after analysis.

**Ethical Consideration:** before any attempt to collect data, approval to conduct the study was obtained from Institutional Review Board (IRB) of School of Medicine, Addis Abba University. Each participant was informed about the purpose of the study, the right to refuse to participate in the study, and anonymity and confidentiality of the information gathered. They were assured that they would not be penalized for not participating and all assessed laboratories were coded for confidentiality purpose.

## Results

### Result from Questionnaire

A total of 213 (100%) laboratory professionals were participated of these 135 (63.4%) were males. One hundred thirty (61%) of the respondents were from government health institutions and remaining were from private health institutions. One hundred thirty one (61.5%) were Bachelor Degree holders and 82(38.5%) had diploma. Sixty one (28.7%) respondents had been working as laboratory or section head, and quality officer and more than 179 (74%) had 3 and above years of working experience in laboratory fields. Laboratory professionals were interviewed about laboratory quality system essentials and accreditation, of them 197 (92.5%) respondents replied that they had knowledge on quality system essentials and concerning laboratory accreditation, 155 (72.8%) of the respondents responded that they had knowledge on accreditation. Among those participants who have information on accreditation 151 (97.4%) believed that laboratory accreditation improves the quality of laboratory services in addition 77 (49.7%) believed that their laboratories have a capacity to be accredited by accreditation body (Table 1).

**Table 1:** Socio-demographic Characteristics and awareness on Quality system essentials and Accreditation of laboratory professionals working in government and private health institutions, Addis Ababa, Ethiopia (n = 213)

Variable	Number/Frequency	Percent (%)
**Sex**		
Male	135	63.4
Female	78	36.6
**Age Group**		
20-30 Years	121	56.8
31-40 Years	59	27.7
41-50 Years	18	8.5
51-60 Years	15	7.0
**Profession**		
Laboratory Technician	82	38.5
Laboratory Technologist	131	61.5
**Educational Level**		
Diploma	82	38.5
Degree	130	61.0
Master Degree	1	0.5
**Working Organization**		
Government	130	61.0
Private	83	39.0
**Working Experience in Laboratory Fields**		
1-2 Years	34	16.0
3-5 Years	61	28.6
6-10 Years	48	22.5
> 10 Years	70	32.9
**Position**		
Laboratory head	20	9.4
Section/Department head	33	15.5
Laboratory expert	152	71.3
Quality Officer	8	3.8
**Knowledge on laboratory quality system essentials**		
Yes	197	92.5
No	16	7.5
**Knowledge on laboratory accreditation?**		
Yes	155	72.8
No	58	27.2
**Information about laboratory accreditation bodies?**		
Yes	107	69.02
No	48	30.98
**Do you think accreditation can improves laboratory services?**		
Yes	92	43.2
No	111	52.1
I don't know	10	4.7
**Do you think accreditation importance for your laboratory?**		
Yes	153	99.6
No	1	0.4
**Do you think high qualified staff can make laboratory accredited?**		
Yes	114	73.55
No	41	26.45
**Does your laboratory have a capacity to be accredited?**		
Yes	77	49.7
No	58	37.4
I don't now	20	12.9
**Do you want more Information about laboratory accreditation?**		
Yes	202	94.8
No	11	5.2

### Laboratory Assessment Results from Checklist

AWHO-AFRO laboratory accreditation assessment checklist was used for assessment of the status of each laboratory. And as part of the checklist, 12 quality system essential elements implementations in each laboratory facility were assessed and discussed separately (Table 2).

**Table 2:** Laboratory facilities scored in each section of quality system essential elements as measured by the WHO-AFRO accreditation checklist.

Institution Code	Ownership of facility	Documents & Records (25)	Management Review(12)	Organization& Personnel(20)	Client Manage. (10)	Equipment(32)	Internal Audit (5)	Purchasing & Inventory(31)	Information Management.(14)	Process Control (43	Corrective Action(8)	Occurrence Management. (10)	Facilities & Safety (40)	Average Score
**1**	Gov	8	8	12	2	13	0	10	12	28	3	2	23	**121**
**2**	Pvt	0	1	1	2	8	0	11	3	6	0	0	8	**40**
**3**	Pvt	4	0	2	0	7	0	7	4	7	0	0	7	**38**
**4**	Gov	12	7	11	1	10	0	19	8	26	3	1	24	**122**
**5**	Gov	5	1	8	0	8	0	14	5	13	1	0	18	**73**
**6**	Pvt	11	8	10	5	21	0	11	8	36	6	10	29	**155**
**7**	Gov	7	3	9	5	11	0	14	9	18	0	0	25	**96**
**8**	Pvt	3	2	2	1	10	0	9	6	6	0	0	19	**58**
**9**	Pvt	9	2	14	4	20	0	25	4	19	4	0	20	**124**
**10**	Pvt	7	1	2	5	7	0	15	6	11	0	1	5	**41**
**11**	Pvt	12	3	11	3	22	1	21	12	20	4	2	23	**134**
**12**	Gov	4	3	10	3	6	0	11	8	12	2	0	13	**72**
**13**	Pvt	5	2	7	4	17	0	16	5	14	2	0	8	**80**
**14**	Pvt	6	1	1	4	14	0	8	7	7	2	0	13	**64**
**15**	Pvt	4	2	1	2	19	0	10	12	14	2	0	8	**74**
**16**	Pvt	3	3	4	5	21	0	11	7	11	0	0	16	**81**
**17**	Pvt	11	3	8	4	21	0	9	9	18	2	0	13	**98**
**18**	Gov	6	2	6	0	2	0	4	3	5	0	0	16	**44**
**19**	Pvt	2	1	2	0	4	0	7	4	4	0	0	13	**37**
**20**	Gov	3	4	12	2	11	0	10	9	16	2	0	12	**87**
**21**	Gov	1	5	9	2	15	0	11	7	18	1	0	24	**93**
**22**	Gov	1	3	7	1	4	0	2	5	3	0	0	6	**32**
**23**	Pvt	0	2	3	0	8	0	12	4	9	0	0	19	**57**
**24**	Gov	9	6	8	4	17	0	20	6	18	1	1	28	**118**
**25**	Pvt	0	2	8	2	8	0	9	4	6	0	0	6	**46**
**26**	Pvt	4	0	4	1	6	0	6	6	5	0	0	8	**40**
**27**	Gov	6	2	4	1	6	0	10	6	10	0	0	15	**60**
**28**	Gov	3	3	3	0	7	0	4	6	11	0	0	16	**53**
**29**	Pvt	0	0	0	3	12	0	10	5	10	0	0	3	**43**
**30**	Gov	0	1	4	2	3	0	10	7	10	0	0	8	**45**

Gov= Government; Pvt = Private

### Documents and Records

Thirty laboratory facilities were assessed to evaluate the document and records practice and this section has 25 points. Accordingly 5 (16.7%) laboratories scored 0 and 23 (76.7%) scored less than 10 with mean score 4.9 and 2(6.7%) laboratory facilities scored 12 (the highest) points.

### Management Review

Three (10%) facility scored 0 point, and majority 24 (80.0%) of laboratory scored between 1 and 6 and 2 (6.7%) laboratories scored 8 (the highest score) out of 12 points with mean 2.7 score.

### Organization and Personnel

Out of 30 assessed laboratory facilities 5 (16.7%) scored more than 10 out of 20 points but 1 (3.3%) laboratory scored 0 point.

### Client Management and Customer Services

Out of 30 assessed laboratories facilities on client management and services, 6(20%) laboratories scored 0 point, and majority 20(66.7%) facilities scored less than 5 (50%) out of 10 points

### Equipment

Equipment part of the checklist had 32 points and out of 30 assessed laboratory facilities 1 (3.3%) laboratory scored the lowest point 2, 1 (3.3%) laboratory facility scored the highest point 22 but majority 15(69.8%) of laboratories scored less than 16 (50%) points.

### Internal Audit

The internal audit section has 5 points and around 97% (29) of the assessed laboratory facilities in internal audit section scored 0, and only 1 laboratory scored 1 (20%) point.

### Purchasing and Inventory

Regarding purchasing and inventory, 1 (3.3%) laboratory scored 2 points (the lowest score) and 1 (3.3%) laboratory also scored 25 points (the highest point) and the mean score was 11.2. Of 28, 15(50.0%) laboratory scored between 3 and 10, 12 (39.6%) scored between 11 and 20, and 1 (3.3%) scored 21. Twenty six (86.7%) laboratories scored less than 55% the minimum requirement points.

### Information Management

Concerning laboratory information management, out of 30 assessed laboratory facilities, 3 (10.0%) laboratories scored 2 (the lowest score) points and 3 (10%) laboratories scored 12 (the highest score) points out of 14 with mean score 6.6. The remaining 15 (50.0%) laboratories scored between 4 and 5, and 10 (33.3%) scored between 7 and 9. Twenty one (70.0%) laboratories scored less than the minimum requirement for accreditation.

### Process Control and Internal & External Quality Assessment

Process control and internal & external quality assessment section has 43 points; out 30 assessed laboratory facilities, 1 (3.3%) laboratory scored 2 points (the lowest score), and 1 (3.3%) facility scored 36 points (the highest score). Twenty five (83.3%)laboratories scored below 20 points.

### Corrective Action

Thirty laboratory facilities were also assessed for corrective action activities and the majority 16(53.3%) scored 0 point, and 1 (3.3%) laboratory scored 6 out of 8 points and the remaining 13 (42.9%) laboratory scored between 1 and 4.

### Occurrence Management

Out of 30 assessed laboratory facilities for occurrence management, 24(80%) facilities scored 0 point and 4(10%) facilities scored between 1 and 2. The remaining facility scored 10 out of 10.

### Facilities and Safety

Under facilities and safety section the assessment results showed that out of thirty assessed laboratory facilities 1(3.3%) laboratories scored 3 points (the lowest score) and 1 (3.3%) scored 29 points (the highest score) out of 40 with mean score 14.9. Of 28(93.3%) laboratories, 22 (73.3%) scored below 20 points.

### Status of Laboratory Sections/Department towards Accreditation

**Figure 1:**
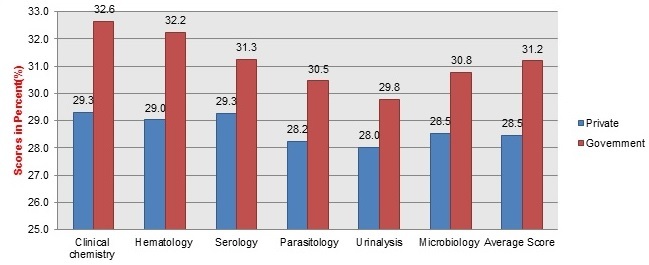
Average scores in percent (%) for each laboratory department based on the ownership of facility as measured by the WHO-AFRO accreditation checklist

**Figure 2:**
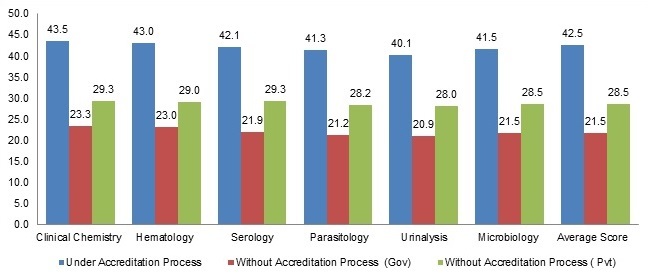
Average scores in percent (%) for each laboratory department based on the accreditation process and ownership as measured by the WHO-AFRO accreditation checklist

Assessment was conducted in six major laboratory sections/departments; such as Clinical chemistry, Hematology, Serology, Parasitology, Urinalysis and Microbiology laboratories including the whole laboratory facility. Results showed that the highest score earned in the laboratory section was 157 (62.8%) and only 1(3.3%) private health facility laboratory scored 157 points in Clinical Chemistry, Hematology, Serology and Parasitology sections which is the minimum required points for accreditation. In addition the microbiology and Urinalysis sections scored 156 (62.8%) and 154 (61.6%) points respectively. Another one private health facility laboratory also scored 138 (55.2%) points in Clinical Chemistry, Hematology, Serology and Microbiology sections which is a minimum required point for accreditation but the remaining 2 laboratory sections scored below the minimum required points (55.2%). In Clinical Chemistry, Hematology, and Serology sections the lowest score was 34 (13.6%) points and the mean scores were 76.9, 76.1, and 75.3 respectively. Regarding Parasitology and Urinalysis laboratories the lowest score were 31(12.4%) with mean score 71.9 and 73.0respectively in addition the lowest score for Microbiology was 32 (12.8%) with mean score 73.8 points. According to the assessment finding, the government laboratory facilities had 81.5(32.6%) the highest average score in Clinical Chemistry section and the lowest average score was from Urinalysis section with 29.8 (74.5%) points. Whereas the private laboratory facilities had 73.3 (29.3%) the highest average score in Clinical Chemistry and Serology sections and the lowest average score was Urinalysis section with 28 (70.0%) points. Six government health facility laboratories were under accreditation process and their highest and lower average scores were108.8 (43.5%) in Clinical Chemistry section and 100.3 (40.1%) in Urinalysis section respectively. However the highest average score for the government laboratory facilities without laboratories under accreditation process was 58.3 (23.3%) in Clinical Chemistry with lowest score 52.3 (20.9%) in Urinalysis section (Figure 1, Figure 2).

### Overall Laboratory Facility Status towards Accreditation

**Figure 3:**
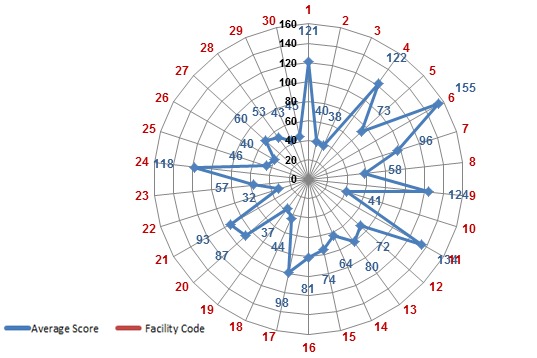
Average scores for each laboratory facility as measured by the WHO-AFRO accreditation checklist

A total of 30 organizations’ laboratory were assessed to see the status towards accreditation as one facility; according to the finding the highest and the lowest scores were 155 (62.0%) and 32 (12.8%) points respectively and the mean score was 74.1 (29.6%). Out of 30 laboratories, only 1 (3.3%) private laboratory scored 155 (62.0%) which is a minimum required point for WHO-AFRO accreditation program and it is eligible to obtain only for star 1 certification (Figure 3). Regarding each section/ department, Clinical Chemistry, Hematology, Serology and Parasitology laboratory sections scored 157 (62.8%) points and Urinalysis and Microbiology laboratories sections scored 154 (61.6%) and 156 (62.4%) points respectively. Each section fulfilled a minimum required point for WHO-AFRO accreditation program and they are eligible to obtain star 1 certification. The general assessment finding showed that 10(33.3%) laboratories scored less than 50 (20%) points, 14 (46.7%) scored between 51 and 100 (21% & 40%) points, 5 (16.7%) scored between 101 and 150 (41% & 60%) points, and only 1 (3.3%) laboratory facility scored 155(62.0%) points. According to the assessment finding, the government laboratory facilities had 78.2(31.2%) mean score of these 6 government health facility laboratories were under accreditation process with the mean score 106.2(42.5%). However the average score for the government laboratory facilities without laboratories under accreditation process was 53.9 (21.5%) while the private laboratory facilities had 71.2 (28.5%) mean scores (Figure 1,Figure 2 and Table 3).

**Table 3:** Each section of laboratory facilities scored as measured by the WHO-AFRO accreditation checklist from 250 points

Lab Code	Ownership of facility	Clinical chemistry		Hematology		Serology		Parasitology		Urinalysis		Microbiology		Average Score	
		Point	%	Point	%	Point	%	Point	%	Point	%	Point	%	Point	%
1*	Gov	122	48.8	122	48.8	122	48.8	120	48	121	48.4	121	48.4	121	48.4
2	Pvt	41	16.4	39	15.6	41	16.4	41	16.4	41	16.4	41	16.4	40	16
3	Pvt	38	15.2	38	15.2	38	15.2	38	15.2	38	15.2	38	15.2	38	15.2
4*	Gov	131	52.4	123	49.2	113	45.2	115	46	104	41.6	114	45.6	122	48.8
5	Gov	75	30	75	30	75	30	70	28	70	28	73	29.2	73	29.2
6	Pvt	157	62.8	157	62.8	157	62.8	157	62.8	154	61.6	156	62.4	155	62
7*	Gov	95	38	98	39.2	95	38	93	37.2	93	37.2	93	37.2	96	38.4
8	Pvt	59	23.6	57	22.8	60	24	57	22.8	57	22.8	57	22.8	58	23.2
9	Pvt	127	50.8	127	50.8	127	50.8	123	49.2	122	48.8	122	48.8	124	49.6
10	Pvt	60	24	60	24	61	24.4	40	16	37	14.8	41	16.4	41	16.4
11	Pvt	138	55.2	138	55.2	138	55.2	134	53.6	134	53.6	138	55.2	134	53.6
12	Gov	83	33.2	80	32	74	29.6	69	27.6	67	26.8	68	27.2	72	28.8
13	Pvt	80	32	80	32	80	32	80	32	80	32	80	32	80	32
14	Pvt	65	26	62	24.8	63	25.2	62	24.8	62	24.8	66	26.4	64	25.6
15	Pvt	73	29.2	73	29.2	73	29.2	72	28.8	73	29.2	74	29.6	74	29.6
16	Pvt	84	33.6	84	33.6	78	31.2	78	31.2	75	30	75	30	81	32.4
17	Pvt	99	39.6	99	39.6	99	39.6	98	39.2	98	39.2	98	39.2	98	39.2
18	Gov	46	18.4	46	18.4	44	17.6	44	17.6	43	17.2	44	17.6	44	17.6
19	Pvt	37	14.8	37	14.8	39	15.6	37	14.8	37	14.8	39	15.6	37	14.8
20*	Gov	89	35.6	89	35.6	89	35.6	87	34.8	86	34.4	87	34.8	87	34.8
21*	Gov	95	38	95	38	95	38	86	34.4	83	33.2	88	35.2	93	37.2
22	Gov	34	13.6	34	13.6	34	13.6	31	12.4	31	12.4	32	12.8	32	12.8
23	Pvt	59	23.6	56	22.4	59	23.6	56	22.4	56	22.4	59	23.6	57	22.8
24*	Gov	121	48.4	118	47.2	118	47.2	118	47.2	115	46	120	48	118	47.2
25	Pvt	45	18	45	18	46	18.4	45	18	45	18	46	18.4	46	18.4
26	Pvt	42	16.8	40	16	40	16	40	16	40	16	40	16	40	16
27	Gov	70	28	68	27.2	59	23.6	62	24.8	61	24.4	62	24.8	60	24
28	Gov	55	22	55	22	53	21.2	53	21.2	52	20.8	55	22	53	21.2
29	Pvt	42	16.8	42	16.8	45	18	42	16.8	42	16.8	43	17.2	43	17.2
30	Gov	45	18	45	18	45	18	42	16.8	42	16.8	43	17.2	43	17.2

Gov= Government; Pvt = Private

* Facilities under accreditation process

## Discussion

Although majority of the laboratory professionals had knowledge on laboratory quality system essentials and accreditation and they believed that the laboratories have a capacity to be accredited, almost all laboratories were not establishing quality management system according to expected standards and they were far from accreditation requirements. Even if most of the laboratory professionals believed in the benefit of accreditation for improvement of laboratory quality services, the practicing of quality system essential elements were poor and as result status of most laboratories towards of accreditation were below the minimum requirement by WHO-AFRO Accreditation Process. In this study majority of laboratories scored less than 50% in the quality system essential elements especially the situations were more worst internal audit, corrective action, occurrence management document and records, management review, organization and personnel, and clients management. This finding was found to be comparable with baseline assessment for accreditation conducted in 24 laboratories in Ethiopia that shows majority of the laboratories scored less than 50% [7], another baseline study done in 12 laboratories in Tanzania was also comparable with our finding, it showed that most of the laboratories scored less than 50% [12]. This outcome could be due to absence of laboratory policy, poor management commitment, poor resource allocation, poor laboratory designing, lack knowledge,and shortage of supplies. The assessment finding also showed us the government laboratories had better mean score(31.2%) than private (28.5%) but if we compare government with private laboratories excluding the laboratories under accreditation process, the government laboratories had lower mean score(21.5%) than private laboratories; it might be due to better infrastructure and management commitment. Those 6 laboratories under accreditation process had 42.5% average score and they were under implementation of quality management system and it helped them to score better results. If the technical supports would be continued, these laboratories could be fulfilled a minimum required point for WHO-AFRO accreditation program than others laboratories in the near future.

It is well documented that implementation of laboratory standards are verified through the process of accreditation and accredited medical laboratories demonstrate a well-functioning quality management system, technical competence, and timely and customer-focused services that contribute to patient care [13]. But in this study an assessment result showed that out of 30 assessed laboratory facilities only one private laboratory was scored the minimum required points for accreditation. This finding is not consistent with study done in Thailand and South Africa which showed that more than 197 in Thailand [14] and 312 laboratories in South Africa [15] were accredited. The reasons could be that Thailand and South Africa had a long time experience in accreditation program. However a survey conducted in 2009 showed that only28 laboratories were accredited in sub-Sahara countries [15], it might be comparable with our finding and the reasons could be due to shortage of resource, lack government commitments, poor attention for laboratory services and shortage qualified personnel. It was very good start to see 6 government laboratories were under WHO accreditation process with technical supports and their average score was greater than the other laboratories. The finding was comparable with that of Guy M et al in Lesotho, where it was found that Mafeteng District Laboratory showed a significance improvement because of accreditation process [13] and another similar findings reported from Tanzania and Thailand also showed that laboratories improved their quality due to accreditation process [12, 16]. Moreover accreditation program in Kenya also showed that significant reduction of client complaints by 82% [10]. Although most of the laboratories did not implement quality management system properly, our finding showed that majority of laboratory professionals believed that accreditation is important for the improvement of laboratory services. However our finding was inconsistent with the finding of Verstraete and colleagues where majority of the participants replied that accreditation is increased workload without considering the benefit of accreditation for the improvement [17]. This thought might be due to lack of knowledge on the quality management system and benefit of accreditation. In general laboratory accreditation verify the adherence of laboratories to established quality management system and competence standards deemed necessary for accurate and reliable patient testing and safety of staff & clients. Furthermore, accreditation is an opportunity for continual improvement of customer service and reduces rates of laboratory errors [18].

## Conclusion

In conclusion, most of the laboratories did not have well established laboratory quality management system and practicing of the quality system essential elements was very limited and almost all laboratories did not have an internal audit system for continual improvement of the quality of laboratory services. Although a quality laboratory testing associated with accreditation is expected to improve patient care by aiding the timeliness and accuracy of medical decision making, the status of laboratories toward of accreditation process were poor and only one laboratory was scored the minimum requirement point for WHO-AFRO accreditation process. As the quality of laboratory services is an essential component for the health care system, the successful implementation of the accreditation process will be bringing a measurable quality improvement in laboratory services and consequently on the healthcare system. Therefore, ministry of health, donors, professional associations and stakeholders should start now to make accreditation of medical laboratories a high priority and should undertake coordinated efforts to integrate accreditation programs into their health policy, planning, and health sector development program. As well allocation enough resources for laboratory infrastructure improvement and endorsement of laboratory policy in Ethiopia are crucial.
